# Engineered nanoparticles induce cell apoptosis: potential for cancer therapy

**DOI:** 10.18632/oncotarget.8553

**Published:** 2016-04-02

**Authors:** Dan-Dan Ma, Wan-Xi Yang

**Affiliations:** ^1^ The Sperm Laboratory, College of Life Sciences, Zhejiang University, Hangzhou, Zhejiang, China

**Keywords:** engineered nanoparticles, cytotoxicity, apoptosis, cancer cells, ENP characteristics

## Abstract

Engineered nanoparticles (ENPs) have been widely applied in industry, commodities, biology and medicine recently. The potential for many related threats to human health has been highlighted. ENPs with their sizes no larger than 100 nm are able to enter the human body and accumulate in organs such as brain, liver, lung, testes, etc, and cause toxic effects. Many references have studied ENP effects on the cells of different organs with related cell apoptosis noted. Understanding such pathways towards ENP induced apoptosis may aid in the design of effective cancer targeting ENP drugs. Such ENPs can either have a direct effect towards cancer cell apoptosis or can be used as drug delivery agents. Characteristics of ENPs, such as sizes, shape, forms, charges and surface modifications are all seen to play a role in determining their toxicity in target cells. Specific modifications of such characteristics can be applied to reduce ENP bioactivity and thus alleviate unwanted cytotoxicity, without affecting the intended function. This provides an opportunity to design ENPs with minimum toxicity to non-targeted cells.

## INTRODUCTION

Nanoparticles (NPs) exist in the natural world as a result of biogenic, geogenic, atmospheric and pyrogenic processes. Examples of natural NPs include geogenic or pyrogenic fullerenes and CNT, biogenic magnetite, atmospheric aerosol, whilst the engineered nanoparticles (ENPs) were either produced inadvertently as by-product of combustion, or were intentionally synthesized to meet the demanding needs of industry, daily life and medicine [1]. With many emerging applications of ENPs, such as those utilized in sporting goods, tires, clothes, sunscreens, cosmetics, foods, diagnostic medicine, imaging and drug delivery [2], the fields related to nanotechnology and nanoscience seem to lead us to the brink of a new industrial revolution. Vastly increasing levels of human exposure to ENP's became already evident and this trend is likely to continue. ENPs are defined as anthropogenic materials with an order of less than 100 nm at one or more dimension. They include those of various shapes and structures ranging from spherical to tubular to irregular and to those that exist as fused, aggregated, or agglomerated forms of organic, inorganic, crystalline or amorphous materials [3]. As the size of the particle decreases, its relative surface area increases and this higher mass-specific surface area renders it more biologically active than larger particles of the same material [4], this brings with it the potential to exert unintentional harmful interactions within biological systems and to the environment. Many new products containing such particles are supplied to the market every year. Common examples include carbon-containing particles, such as carbon nanotubes, functionalized nanotubes, fullerenes, as well as metal containing particles such as TiO_2_, SiO_2_, silver, copper, iron, and gold [3].

ENPs dispersed into air, water, food, clothes or pharmaceuticals may enter the human body through inhalation, uptake *via* skin, ingestion, or injection and readily travel through the body in the blood vessels to be deposited in target organs such as liver, heart, kidney, spleen, bone marrow and other sites where they may trigger injurious responses [5]. Travelling through the respiratory tract is the primary entrance of nanomaterial exposure. Some inhaled ENPs can be deposited at the nasal cavity and bronchus and then be excreted by the body. However, some others, those of a size ranging from 5-50 nm, are more likely to be deposited at the alveoli. When they exceed the phagocytic capacity, this can lead to their travel across the alveolar epithelium, and even the alveolar-capillary barrier, with the potential to cause adverse effects on extrapulmonary tissues such as the heart, liver, and brain. Such exposure may eventually trigger cardiovascular diseases as well as other central nervous system effects [6]. Skin uptake is another primary pathway of ENP exposure, particularly where nano-level particles have been used in cosmetics or clothes. Opinions differ in the potential of solid NPs to overcome the skin barrier, to penetrate the stratum corneum and to diffuse into underlying structures. ZnO nanoparticles, which are widely used in sunscreen products, are reported to possess the ability to penetrate into the viable epidermis *in vivo*. When this occurs they mainly localize at the furrow-cellular border or even penetrate into the skin strata [7]. Although *in vivo* toxicity studies have failed to reveal any readily apparent cytotoxicity, genotoxicity, photo-genotoxicity, general toxicity or carcinogenicity associated with insoluble TiO2 and ZnO nanoparticles, as found in personal care products [8], one should not neglect the possibility of long-term effects. Related to ingestion, NPs contained in food or water or were used in cosmetics or drugs can be directly ingested *via* the gastrointestinal tract and thus enter into the blood stream. While many of these NPs can be excreted through urine, others may lead to significant complications.

Potential health risks of ENPs next to their accumulation in the body arise with their capacity to pass through the blood-brain barrier (BBB). This is the reason why ENPs have been considered as drug carriers for the treatment of central nervous system (CNS) disorders. Their efficacy has been demonstrated in models such as those related to neurodegenerative diseases, neuroregeneration, and brain tumors [9]. The adverse effects of ENPs on the functioning of the CNS have also been reported. Studies by Campbell et al. (2005) using ovalbumin-sensitized BALB/c mice exposed to airborne fine and ultrafine ambient particles showed increased levels of pro-inflammatory cytokines interleukin-1 alpha (*IL-1a*) and tumor necrosis factor alpha (*TNF-a*) as well as the immune-related transcription factor *NF-kB* in the brain [10]. This indicates the occurrence of ENP induced pathological changes with the potential to induce neurodegenerative disease. Similarly, a series of NPs have been reviewed as being able to penetrate the blood-testis barrier and affect spermatogenesis [11]. This probably is the mechanism of NPs inducing damage of the tight junction of the Sertoli cells, as reported by Zhang et al. (2015). In the latter study exposure to silver nanoparticles (AgNPs) of 10 and 20 nm resulted in a decrease in mRNA levels of tight-junction related genes in the mice Sertoli cells [12]. Significantly, some nanoparticles have also been reported to be able to cross through the blood-fetus barrier resulting in malformed fetuses among mice offspring [13].

There are many kinds of manufactured nanomaterial products including TiO_2_, ZnO, CeO_2_, Fe_2_O_3_, and CuO (as metal oxide nanoparticles) as well as gold, silver, platinum and palladium (as metal nanoparticles), and other carbon based ENP's such as carbon nanotubules (CNTs) and quantum dots [14]. These have been widely and increasingly used by the industry as catalysts, fuel, cosmetics and food. They have also been applied in nanomedicine and bio-imaging. Although such an increase in use has aroused people's awareness of toxic effects of some ENPs, the mechanism of ENP toxicity is, as yet, not well understood. As apoptosis has been reported in a variety of cell types *via* contacting with various ENPs, this paper will discuss in detail the mechanisms by which ENPs exert apoptosis. This will include the way they enter cells, their distributions in subcellular regions, and the way they change cellular morphology. We also highlight the specific molecules involved in such processes towards a better understanding of ENP cytotoxicity. We hope this may provide a more comprehensive perspective to evaluate the safety of nanoparticles. This review provides a new possibility in cancer treatment. In recent years, the number of cancer patients is increasing substantially. Almost each cancer treatment has its own side effects, and its curative ratio is very low. ENPs, when modified or loaded with signal molecules, can selectively target the cancer cells and induce cell apoptosis there, especially in organs that have special barriers, such as brain and testes. Therefore, the ENPs induced cancer therapy may become a trend in the future.

## UPTAKE OF ENPS AND THEIR INTRACELLULAR LOCALIZATIONS

Cell membranes consist of different receptors that function in cell-cell signaling, cell adhesion, interaction and the immune recognition of foreign substances. Several ENPs have been reported to bind directly to surface receptors and occupy specific residence times in those locations. Oligonucleotide-conjugated gold nanoparticles (AuNP-ssO), for example, were found attached as single nanoparticles to the plasma membrane of bovine sperm [15]. This resulted in decreased amount of free thiol groups on the surface of the membrane, affecting sperm functionality and causing detrimental effects such as loss in motility by interfering directly with membrane bound ion transporters. According to Hirano et al. (2008), multi-walled carbon nanotubes (MWCNTs, 67 nm) triggered cytotoxic effects in mouse macrophages by reacting with the macrophage receptor collagenous structure (MARCO) and rupturing the plasma membrane [16]. Some other ENPs are also retained in the membrane fraction due to its relatively large primary particle size. Compared to nano-titanium dioxide (TiO_2_, 24.0±7.2 nm), a large fraction of nano-hydroxyapatite (Nano-HA, 51.1±12.1 nm) were found at the cellular membrane of the human oral epithelium where it was seen to cause elevated reactive oxygen species (ROS) levels and apoptotic profiles [17].

A number of techniques have been applied to study the biodistribution of ENPs in cells. Immunogold labeling, FITC-tagging, and transmission electron microscopy (TEM) are the most frequently used techniques. Results showed that ENPs were taken up by animal cells through a variety of ways, including direct penetration, caveolae dependent endocytosis, lipid raft composition, clathrin-dependent endocytosis or phagocytosis [18]. The direct ENP penetration refers to either entering the membrane by passive diffusion, or by non-specific permeation as a result of ENPs-inflicted membrane damage [19]. The active energy-taking uptake of ENPs include different endocytosis vesicles with different sizes: macropinosomes 1-5 μm in diameter, clathrin mediated endosomes up to 120 nm, caveolae 50-80 nm [20]. Upon uptake, the intracellular localization and exocytosis depend on ENPs sizes, surface characteristics and the ability to form aggregates, etc. [18]. ENPs internalized into mammalian cells have been frequently found to be co-localized in the endosomes or lysosomes, either for degradation or recycling. Some others were observed to be randomly dispersed in the cytoplasm or interact with the nuclear membrane or organelles and were often seen to be causing various adverse effects to cells.

Among all the reports of ENP associated organelle damages, mitochondria seem to be the most sensitive target. Uptake of silver nanoparticles (AgNPs) by normal human lung fibroblasts (IMR-90) and human glioblastoma cells (U251) occurs mainly *via* endocytosis with a uniform intracellular distribution of AgNPs in the cytoplasm, nucleus, and nucleoli. AgNPs treated cells showed autophagic vacuoles filled with mitochondria-like structures as well as other unknown contents and also exhibited chromosome instability and mitotic arrest [21]. Damaged mitochondria were also found in mouse TM3 Leydig cells and the TM4 Sertoli cells when treated by 10 nm and 20 nm AgNPs for 24 hours. Such observations were accompanied by cell autophagy and apoptosis [22]. Bressan et al. (2013) studied the interaction of AgNPs with human dermal fibroblast cells and found that the internalized AgNPs were closed to the outer membrane of the mitochondria. This caused direct mitochondrial damage and the disturbance of respiratory chain function with a result of increased ROS level and oxidative stress [23]. Cerium dioxide nanoparticles (CeO_2_ NPs), with diversified industrial uses and novel therapeutic applications, were also studied in human peripheral blood monocytes. TEM analysis showed that human peripheral blood monocytes, upon treatment with 10 μg/mL CeO_2_ NPs for 40 h, resulted in enlarged or swollen mitochondria and included the induction of apoptosis and autophagy accompanied by the accumulation of aggregated CeO_2_ NPs in vesicles and free CeO_2_ in cytoplasm [24]. Mitochondrial swelling, nuclear shrinkage, chromatin condensation and the evacuation of lamellar bodies were also found in mice pneumonocytes when they had been engulfed in nanosized titanium dioxide (nano-TiO_2_), where the classical morphology characteristics of cell apoptosis also appeared [25].

Along with mitochondria, the endoplasmic reticulum (ER) and the nucleus also appear to be targeted by ENPs. Zinc oxide nanoparticles (Nano-ZnO), which are widely used in daily commodities, have been reported to induce hepatotoxicity through ER stress. ER swelling and ribosomal degranulation were also observed by TEM in the liver tissues treated with Nano-ZnO [26]. Obvious ER aggregation and the associated swelling were also observed in nano-ZnO treatment of human umbilical vein endothelial cells (HUVECs) [27]. The human hepatic cell line L-02 also showed cellular swelling, microvilli disappearance, mitochondrial vacuolization and ER ectasis after incubation with 0.6 mg/mL of 21 nm SiO_2_ colloids for 24 h [28]. In addition to the ER, nano-SiO_2_ was also reported to be deposited in the nuclear membrane and cause its deterioration in human embryonic kidney cells HEK-293 when treated with 100 or 200 mg/L nano-SiO_2_ for 48 h. This induced cell apoptosis through oxidative-stress-mediated DNA damage and p53 activation [29].

**Figure 1:**
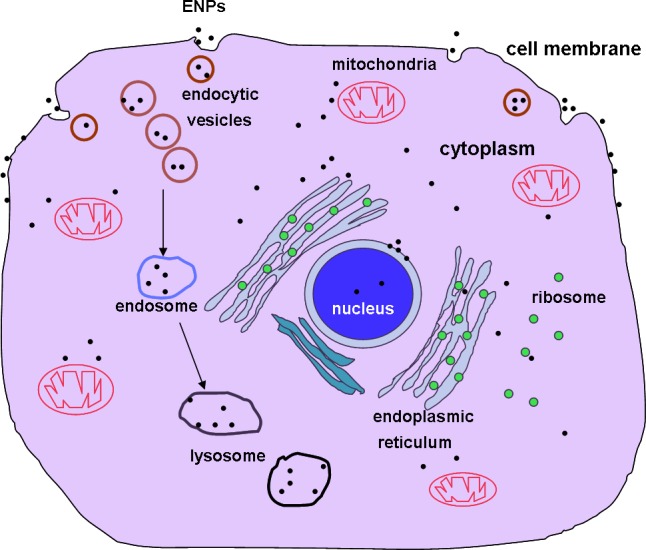
Uptake of ENPs and their intracellular localizations ENPs are taken up by animal cells through pinocytosis, caveolae dependent endocytosis, lipid raft composition, clathrin-dependent endocytosis or phagocytosis. ENPs are distributed on the cell membrane, in the endosomes or lysosomes, randomly dispersed in the cytoplasm, interacting with the nuclear membrane, in mitochondria or in the endoplasmic reticulum.

To summarize, the function and action of ENPs on cells depend on a variety of factors including whether the particles are aggregated or in free form, particle size, target cell type, length of ENP treatment, as well as the inner subcellular distribution of the ENPs and organelles affected. Some ENPs deposited on the cell membrane while others were engulfed into cells (Figure 1). For those entering cells, some were uniformly dispersed inside the cells, others were deposited in endosomes or lysosomes and some induced cell apoptosis through damaging the mitochondria. Others caused similar damage by targeting the ER, nuclear membrane or chromatin.

## ENP INDUCED CELL APOPTOSIS AND THE PATHWAYS INVOLVED

The nano-sizes of these particles make them easy to be transported into biological systems, thus having the potential to have many effects on susceptible organisms. Changes in gene expression, abnormal apoptosis, enhanced oxidative stress and pro-inflammatory effects involving ENPs exposure have been reviewed by Magaye et al. (2012) [30]. The *in vitro* responses that ENPs elicit on different cells also differ considerably which may correspond to similar factors of size, shapes or agglomeration aspects of the ENPs, and ENP types, surface modifications, surface charges, doses, etc. All such factors can influence ENP pathways in treated cells. Here we introduce several mechanisms involved in ENP actions in inducing abnormal cell apoptosis (Table 1).

**Table 1:** ENPs induced cell apoptosis in different cell lines

ENPs type	ENPs size	Induced cell apoptosis	Mechanism	Reference
Nano-TiO_2_	5-6 nm	mice lung tissue	Oxidative stress	[31]
AgNP	25-70 nm	human lung fibroblast (HLF) cell	mitochondrial dysfunction, ROS	[32]
NiONP	≤50 nm	human bronchial epithelial cells (BEAS-2B)	SIRT1, caspase3	[33]
PAMAM	45 nm	human lung cells WI-26 VA4	Intrinsic mitochondrial pathway	[34]
MWCNOs and MWCNTs		human skin fibroblasts cell (HSF42)	Altered gene expressions	[35]
Nano-SiO_2_	15 nm, 30 nm	human keratinocyte HaCaT cells	oxidative stress	[36]
Nano-TiO_2_	21 nm	HaCaT cells	oxidative damage, intrinsic mitochondrial pathway	[37]
Cr_2_O_3_ NPs	26.5 nm	HaCaT cells	oxidative DNA damage, caspase 3	[38]
NiNPs	92.32 nm	mouse epidermal JB6 cells	Casepase8/AIF	[40]
Nano-SiO_2_	15 nm	PC12 cells	oxidative stress	[42]
Nano-SiO_2_	20 and 50 nm	PC12 cells	oxidative stress	[45]
Nano-TiO_2_	21 nm	PC12 cells	oxidative stress	[46]
AgNP	14 nm	PC12 cells	Caspase8/9	[47]
CeO_2_ NPs	10-30 nm	monocytes	mitochondria/AIF	[51]
Nano-TiO_2_	5-6 nm	mice spleen	Oxidative stress, ER stress	[52]
Nano-TiO_2_	25 nm	human lymphocytes	JNK/p38-caspase 8-Bid pathway	[53]
CoO-NPs	62±4 nm	human lymphocytes	TNF-α-caspase 8-P38-caspase 3	[54]
PS-NPs	20 nm, 40 nm	Caco-2 cells	Oxidative stress	[56]
AgNPs	20±2 nm	Caco-2 cells	Oxidative stress, ER stress	[57]
Nano-ZnO	80 nm	Mice liver	Oxidative stress, ER stress	[58]
Nano- SiO_2_	21 nm	hepatic cells L-02 cells	Oxidative stress, p53, Bax	[28]
Nano-TiO_2_	13-35 nm	human embryonic kidney cell line HEK-293	Oxidative stress, p53, Bax, caspase 3	[29]
Nano-Cu	15 nm	podocytes	Oxidative stress	[60]
Nano-Cu	25-40 nm	Mice kidney	Oxidative stress, intrinsic/extrinsic pathway	[61]
ZnO NPs	42 nm	human umbilical vein endothelial cells (HUVECs)	ROS, ER stress	[27]
MWCNOs	31.2 nm	HUVECs	ROS, DNA damage	[63]
SWCNTs		Rat aorta endothelial cells(RAECs)	ROS, mitochondrial pathway	[64]
Nano-TiO_2_	50-100 nm	Mice testes	FasL, p38, caspase-3	[65]
AgNPs	10 nm, 20 nm	Male leydig cell (TM3) the Stertoli cell (TM4)	Caspase 3/8/9	[12]

### ENP induced pulmonary injury

As mentioned, ENPs that are taken in mainly through inhalation tend to lead to increasing concerns about effects on pulmonary health in particular. Nasal administration of nano-TiO_2_ (5-6 nm by TEM) to mice for 90 consecutive days at 10 mg/kg BW, for example, resulted in the deposition of nano-TiO_2_ in the lungs. This was accompanied by significant pyknosis of the nucleus, mitochondrial swelling and evacuation of the lamellar bodies, all of these being classical morphological aspects of apoptosis [31]. Microarray analysis revealed significant alterations of the 847 genes in the nano-TiO_2_ treated mouse lung tissues. Such alterations resulted in examples of apoptosis, oxidative stress, changes to the cell cycle and alterations in cell proliferation. In such cases, lung apoptosis may be caused by the elevated production of reactive oxygen species (ROS), as demonstrated by peroxidation of lipids, proteins and DNA in mouse lung tissue. Monodispersed AgNPs with a range of 25 to 70 nm were able to cause human lung fibroblast (HLF) cell apoptosis at concentrations of 250 μg/mL for 72 h, with 25 nm AgNPs causing the highest apoptosis rate (80%). This implies that nano-AgNP cytotoxicity is increased with the decrease of particle size [32]. The nano-AgNPs taken up by cells may release Ag ions, the elevated Ag ions then caused mitochondrial dysfunction and the generation of excessive ROS. This leaves the cells under oxidative stress which subsequently results in cell apoptosis. The wide application of nano-sized nickel (Nano-Ni) compounds offers another occupational hazard that is associated with pulmonary injury. NiO nanoparticles (NiONPs), of sizes smaller than 50 nm, were able to enter human bronchial epithelial cells (BEAS-2B) and release Ni^2+^ inside the cells. This resulted in inhibited cell viability through an apoptotic process as indicated by activated caspase-3 and the increased numbers of Annexin V positive cells [33]. It was also found that sirtuin 1 (SIRT1), an NAD-dependent deacetylase the substrates of which include p53, Foxo and Ku70, was suppressed by the intracellularly released Ni^2+^. This rendered the cells more sensitive to apoptosis. SIRT1 displays essential inhibitory effects by directly deacetylating the C-terminal lysine residue of p53, thus inhibiting the p53-dependent apoptosis pathway. The dendrimer nanosized polyamidoamine (PAMAM), widely used in clinical settings, has been also reported to exert cytotoxicity on human lung cells (WI-26 VA4) [34]. The 45 nm PAMAM was found co-localized with mitochondria. This disrupted mitochondrial membrane potential and caused a release of cytochrome c, activating caspases 3 and 9. Cell apoptosis, as demonstrated by annexin V/propidium iodide staining and DNA fragmentation, then occurred. Also, such examples highlight the strong requirement that nanomaterials should be carefully tested and cautiously used from a safety perspective.

### ENPs induced skin injury

In addition to the pulmonary system, which is often directly exposed to airborne ENPs, human skin is another potential target for ENPs, particularly those used in consumer products such as clothes and cosmetics, both of which are designed to have direct contact with skin. A human skin fibroblast cell (HSF42) population, for example, was used to test the cytotoxicity of multiwall carbon nano-onions (MWCNOs) and multiwall carbon nanotubes (MWCNTs) [35]. HSF42 cell cycle arrest and apoptosis/necrosis were seen to be induced by MWCNOs (6 and 0.6μg/mL) and MWCNTs (0.6 and 0.06μg/mL), the effects of which included the activation of genes involved in cellular transport, metabolism, cell cycle regulation and stress response. Nanometer silicon dioxide (nano-SiO_2_) of 15 nm and 30 nm was similarly observed to induce human keratinocyte HaCaT cell apoptosis in a dose-dependent manner. Again, in cases of identical doses, it was observed that the smaller the particle size, the severer was the cell apoptosis [36]. Two dimensional differential gel electrophoresis (2D-DIGE) and mass spectrometry (MS) were used to detect the differences in protein expression. Sixteen proteins in 5 categories were found to display significant changes. The changes in the proteins were associated with oxidative stress. In this study, though apoptosis was thought to be involved in the toxic mechanisms related to nano-SiO_2_, the specific apoptosis signals and how nano-SiO_2_ induces cytotoxicity exactly, remain unclear. In another case, HaCaT apoptosis was induced by 21 nm nano-TiO_2_ where oxidative damage was caused by the generation of ROS and proved to result in mitochondrial permeability transition (MPT) pore opening. This led to the release of cytochrome c and other factors that activated caspase-3 and initiated the pathway of cell apoptosis [37].

Released of soluble metal ions are considered responsible for some of the aspects of cytotoxicity of metal nanoparticles. The HaCaT cellular uptake of Chromium (III) oxide nanoparticles (Cr_2_O_3_ NPs) with a size of 26.5 nm was observed by TEM [38]. The internalized Cr_2_O_3_ NPs formed aggregates and were dispersed in the cytosol. This released soluble Cr (VI) or Cr (III) to the cell interior which increased the intracellular ROS level and reduced the GSH level. Results included oxidative DNA damage and the triggering of the caspase 3-dependent cell apoptosis pathway. Nickel is also widely used in many industries. Workers exposed to a variety of nickel compounds have been reported to exhibit various pathological effects including skin allergies, lung fibrosis and lung cancer [39]. Mouse epidermal JB6 cells exposed to metallic nickel nanoparticles (NiNPs) (92.32 nm) displayed cell apoptosis after a 24 h treatment to a dose range of 0.1-20μg/cm [40]. It was found that NiNPs activated the proapoptotic factors including Fas (CD95). Here, Fas-associated proteins with death domain (FADD), death receptor 3 (DR3) as well as pro-caspase 8, are bound together to form a death including signaling complex (DISC) which has been implicated as an extrinsic apoptotic pathway. In addition, apoptosis-inducing factor (AIF) was also up-regulated by NiNPs and released from the mitochondria to the cytoplasm, entered the nucleus, and caused DNA breakage. This implied a caspase 8/AIF mediated apoptosis pathway [40].

### ENPs induced CNS injury

ENPs have been increasingly used in diagnosis, imaging and drug delivery to the central nervous system. The main reason for this lies in their ease of transport through the blood brain barrier (BBB). Whilst the mechanism of ENPs transport cross BBB has not been fully elucidated, the most probable transport pathway seems to be the lipid-mediated endocytosis of small molecules, together with other blood plasma components. The ENPs may interact with the Low Density Lipoproteins (LDL) on the endothelial cells and then get internalized. The ENPs can also get into the CNS by completely bypassing the BBB at the median eminence, lamina terminalis, or the area postrema [41]. However, this factor becomes very limited if using particles of larger sizes. Little is known about the potentially adverse effects of the ENPs on the brain. In rats, intranasal instillation of SiO_2_-NPs (15 nm by TEM) for 1 day and 7 days result in a significant accumulation in the striatum and the hippocampus [42]. Such a deposition resulted in oxidative stress, inflammation and depleted dopamine in the striatum and in a down-regulation of the tyrosine hydroxylase protein. All such factors increase the risk towards the development of neurodegenerative disorders. In order to study the SiO_2_-NPs induced pathway and mechanism of *in vivo* damage, *in vitro* studies were also carried out through co-incubation model of dopaminergic neuron PC12 cells and SiO_2_-NPs. The PC12 cell is a cell line derived from the rat adrenal medulla pheochromocytoma that includes the typical characteristics of dopaminergic neurons in both form and function. It has therefore been widely used as a paradigm for neurobiological studies [43]. The exposure of PC12 cells to SiO_2_-NPs for 24 h resulted in cell apoptosis accompanied by increased intracellular ROS levels and oxidative damage measured by glutathione (GSH) depletion, methane dicarboxylic aldehyde (MDA) production and superoxide dismutase (SOD) inhibition. Oxidative stress may be the main route of SiO_2_-NPs induced P12 cell apoptosis as brain cells have been reported to be particularly vulnerable to oxidative damage due to their high metabolic rates, high content of cellular lipids and proteins and low level of endogenous scavengers [44]. Oxidative mechanisms also contribute to nano-SiO_2_ (20 and 50 nm) and nano-TiO_2_ (21 nm) induced P12 cell apoptosis, for both NPs this happened in a dose-dependent manner [45–46]. Such brain cell vulnerability was further shown in a previous study using the pretreatment of N-(mercaptopropinyl)-glycine (N-MPG), a ROS scavenger, which inhibited the nano-TiO_2_ induced P12 cell apoptosis to some degree [46].

14 nm AgNP was also shown to have potential neurotoxicological effects [47]. Female rats treated with 4.5 and 9 mg AgNP/kg bw/day for only 14 days showed a decreased level of dopamine concentration, conversely, after 28 days the levels increased, which suggests exposure/time-dependent effects. As disturbances of dopamine levels have been linked to Parkinson's disease [48], an *in vitro* exposure model was conducted to look for the underlying toxicity mechanism. P12 cell apoptosis, as induced by AgNP exposure, was assumed to be caused by the release of Ag ions as no AgNPs were found by TEM within cells. Co-incubation of AgNPs with caspase 8 or 9 inhibitors abolished this AgNP-induced cell apoptosis. This suggests that both the mitochondrial and the death receptor pathways play a role in this cytotoxic mechanism.

It should be noted that pregnant mice exposed to ENPs also gave birth to fetuses with retarded brain development [49]. This indicates the ability of such ENPs to cross the blood-fetus barrier. In one case where pregnant mice were injected 100 μg nano-TiO_2_ (2570 nm) on gestational days, the brains of newborn pups showed altered expression in apoptosis genes at early ages and changed the expression of genes responding to oxidative stress as analyzed by cDNA microarray and Gene Ontology (GO). The expression of genes relating to the function of the CNS was also affected. This could pose a threat to the normal development of the CNS in the young generation [49].

### ENP induced immune system injury

Monocytes work as integral components of the immune system. They play roles in producing antibodies, resisting pathogen invasion and antagonizing diseases. They also act as links between innate and adaptive immune components and are precursors of important antigen-presenting cells such as macrophages and dendritic cells [50]. The increased cytotoxicity of monocytes can lead to impaired early immune responses to foreign agents or infections. This poses concerns in terms of human health. Cerium dioxide nanoparticles (CeO_2_ NPs), with their diverse industrial uses and actively pursued therapeutic applications, were toxic to primary human monocytes at relatively low doses [24]. Monocytes exposed to 5 and 10μg/mL CeO_2_ NPs (range from 10 to 30 nm) for 40 h showed significant apoptosis and DNA fragmentation. An increase in mitochondrial depolarization and the number of cells with activated forms of the pro-apoptotic protein Bax were observed after CeO_2_ NPs exposure. Here, it was not the damaged mitochondria that initiated the caspase pathway, but the apoptosis inducing factor (AIF) that induced DNA fragmentation and subsequent cell apoptosis. In addition, p53 modulated autophagy was also assumed to be part of the CeO_2_ NP toxicity to monocytes.

The spleen is not only an organ working as the body's largest blood filter, it also plays an important role in the immune system and shares responsibility for a number of other areas including the protection of the body from invading pathogens, the recognition and removal of unwanted, damaged, or aberrant cells, the avoidance of tumor formation and the mitigation of various other kinds of diseases [51]. Nano-TiO_2_ (2.5, 5, 10 mg/kg BW), ranging from 5 to 6 nm, as intragastrically administrated to mice for 90 consecutive days, resulted in spleen injury. Increase in spleen indices, immune dysfunction, and severe macrophage infiltration was noted as well as apoptosis in the mouse spleen after Nano-TiO_2_ exposure. Microarray assay results showed that, in addition to the clear expression of indicators of abnormal spleen functions, genes involved in oxidative stress (*Cyp2e1, Mt1, Mt2, Sod3*) were also up-regulated. This resulted in the up-regulation of *Atf4* and *Chac1* as well as in the down-regulation of *Dnajb2*, *Hspa8*, *Hsp90B1* and *Asns*. Such factors further triggered the endoplasmic reticulum (ER) stress load and apoptosis in the splenocytes. Such results call for caution when applying Nano-TiO_2_ in food industry, cosmetic, sunscreens, and in medicine [52].

Nano-TiO_2_ (25 nm) also causes apoptosis in human lymphocytes, as demonstrated by the increase in the sub-G1 cell fractions [53]. The collapse of mitochondrial membrane integrity and the activated caspase 3/9, followed by Poly (ADP ribose) polymerase (PARP) cleavage, indicated that the nano-TiO_2_ induced apoptosis in lymphocytes occurred through an intrinsic mitochondria-dependent pathway. This same pathway is also regulated by caspase 8. When activated, caspase 8 cleaves its substrate Bid. Truncated-Bid (t-Bid) then translocates from the cytosol to the mitochondria and induces a mitochondrial collapse. In this study, it was found that two members of the mitogen-activated protein kinase (MAPK) family, p38 and cJun N-terminal kinase (JNK), play a role in the regulation of nano-TiO_2_ induced lymphocyte survival by activating caspase 8. The metal CoO-NPs exert toxicity to human lymphocytes *via* either direct uptake into the cells or through the release of Co^++^ into the cytoplasm. In such a study, an elevated ROS was attributed to the CoO-NPs (62±4 nm by SEM) induced lymphocyte apoptosis. ROS acts as a critical signaling molecule in the induction of apoptosis through different pathways. On one hand, the ROS induces cellular toxicity like damage to the cell membrane, as reflected by increased lactate dehydrogenase (LDH) levels. On the other hand, excessive ROS induces the activation of the pro-inflammatory cytokine TNF-α, which is followed by the phosphorylation of p38 and the activation of caspase 8 and caspase 3, thus initiating the signaling pathway of apoptosis. *In vivo* studies in mice show that CoO-NPs possesses significant toxicity to peripheral blood mononuclear cells (PBMCs) and platelets as shown after 30 days of exposure starting from 200μg/kg BW. In this, an increase in the level of pro-inflammatory cytokine TNF-αand a decrease in anti-inflammatory cytokine IL-10 was noted as indicators of inflammation [54]. CoO-NPs were, therefore, proven to be toxic to the human primary immune system.

### ENPs induced intestine, liver and kidney injury

Various ENPs are used in the food sector for improving food packaging materials, efficient nutrient delivery and for formulations with improved bioavailability. This makes consumer exposure to ENPs inevitable. Upon ingestion, ENPs reach the gastrointestinal tract and remain there for 4-8 h [55]. Differentiated human colorectal adenocarcinoma cells, Caco-2, were applied to test the toxicity of commonly used polystyrene (PS) nanoparticles (of 20 and 40 nm diameters) in the intestinal membrane. Phosphatidylserine externalization, reduced mitochondrial transmembrane potential and increased caspase activity were all observed along with PS-induced Caco-2 cell apoptosis. The addition of catalase (CAT), an enzyme that selectively decomposes hydrogen peroxide (H_2_O_2_), resulted in significant decrease in apoptosis. This indicated an oxidative stress-induced apoptosis pathway was in operation in such cases. Ingested non-biodegradable nanoparticles represent a particular potential health risk to the intestinal membrane [56]. AgNPs are frequently used in the food sector. One study applied whole-genome mRNA expression to study the effects of four sizes of AgNPs (20±2 nm, 34±3 nm; 61±5 nm; 113±8 nm) exposure on Caco-2 and M-cells. After exposure for 4 h *in vitro*, the AgNPs induced clear changes in the expression of stress responding genes including those associated with oxidative stress, endoplasmatic stress responses, and apoptosis, with cell viability being significantly reduced by 37.5μg/mL AgNPs (20±2 nm) exposure [57].

The oral intake of ENPs may also cause liver injury in organisms. For example, in an *in vitro* study in which mice were gavaged with 200 mg/kg or 400 mg/kg of Nano-ZnO (80 nm) once a day for consecutive 90 days [58], liver injury was observed with focal hepatocellular necrosis and congestive dilation of central veins as well as increased levels of the liver function enzymes alanine transaminase (ALT) and aspartate transaminase (AST). TEM results also showed ER swelling and ribosomal degranulation in the liver tissue. Oral administration of nano-ZnO resulted in the production of MDA and the depletion of GSH. This suggested an imbalance in oxidative status. Apoptosis-related genes (*bax* and *chop*) and proteins (caspase 3/9/12 and JNK) were seen to be highly expressed. This was consistent with the conclusion of oxidative stress promoted by nano-ZnO administration. Whilst C/EBF-homologue protein (CHOP), cJun N-terminal kinase (JNK), and caspase 12 appeared as ER-stress mediated proteins, the liver injury caused by long term oral exposure to nano-ZnO was attributed to oxidative stress and ER stress induced apoptosis. *In vitro* experiments using normal human hepatic cells (L-02) to test the cytotoxicity of nano-SiO_2_ were also conducted. Here the L-02 cells were treated with 0.2, 0.4 or 0.6 mg/ml of 21, 48 or 86 nm SiO_2_ colloids for 24 h. Cell apoptosis was then evidenced using annexin V-FITC/PI double-staining which revealed apoptosis occurring in a dose and time dependent manner. The 21 nm SiO_2_ caused a dose-dependent increase in ROS and lipid peroxidation and a decrease in GSH levels, all of which were indicative of oxidative stress. The oxidative stress induced DNA damage (as shown by the DNA ladder in current study) triggered cell apoptosis by up-regulating p53 and Bax. This explains the mechanism of its toxicity in liver cells [28].

ENP-induced renal injury has also occurred, as noted in recent literature. Just as in the case of nano-SiO_2_, ROS mediated oxidative stress, activated p53, Bax and caspase 3 also all played a role in nano-TiO_2_ (13-35 nm) induced cell apoptosis in the human embryonic kidney cell line (HEK-293) [29]. In addition to nano-TiO_2_, nano-copper of different sizes was proven to induce oxidative stress and apoptosis in kidney cells in both *in vivo* and *in vitro* experiments. Podocytes act as a glomerular filtration barrier whose injury correlates to renal dysfunction [59]. Adult mouse podocytes having been exposed to 1, 10 or 30 g/mL nano-Cu (15 nm) for 3 h displayed cell shrinkage, nuclear condensation and fragmentation indicative of apoptosis [60]. Pretreatment with N-MPG (a ROS scavenger) inhibited the podocyte apoptosis induced by nano-Cu. This suggests that apoptosis occurs *via* an oxidative stress-induced apoptosis pathway. In another *in vivo* study where mice were orally gavaged with 200, 413 or 600 mg/kg BW nano-Cu (25-40 nm) for 3 days, they also investigated the specific apoptosis pathways. It was found that, in addition to intracellular ROS and NO generation, changes in Bcl2/Bax, disruption of the mitochondrial membrane integrity, release of cytochrome c from the mitochondria to the cytosol and the activation of caspase 3 and 9 were all involved in the intrinsic mitochondrial-induced apoptosis pathway. The activation of Fas, caspase 8 and tBid were also suggestive of an extrinsic apoptotic pathway [61].

### ENPs-induced cardiovascular injury

Certain environmental epidemiological studies have correlated atmospheric particles to cardiovascular diseases [62]. In addition to the liver injury mentioned previously, nano-ZnO exposure is also correlated with cardiovascular abnormalities. The *in vitro* exposure of human umbilical vein endothelial cells (HUVECs) to non-cytotoxic concentrations of ZnO NPs (42 nm) resulted in caspase-12 mediated ER stress. This could be used as a sensitive and early biomarker of cell apoptosis. Where ER stress may be caused by ROS induced oxidative stress, the pretreatment of HUVECs with N-acetyl-L-cysteine (NAC, another ROS scavenger) could act to utterly abolish such ER-stress [27]. The HUVECs also underwent apoptosis after 1 and 5μg/mL MWCNOs (31.2 nm) exposure for 24 h where ROS generation and DNA damage were assumed to have caused the apoptosis [63]. While SWCNTs under concentrations of 100μg/mL were suggested to be safe for drug delivery or other use, exposure to 200μg/mL SWCNTs after 48 or 72 hours was found to induce apoptosis in rat aorta endothelia cells (RAECs) and was accompanied by chromatin condensation, internucleosomal DNA fragmentation and caspase 3 activation [64]. The increase of ROS levels and the reduction of GSH in SWCNT-treated RACEs resulted in DNA-protein crosslinks, a kind of DNA damage not easy to repair, which activated p53, TNF-αand Bax and caused cell apoptosis *via* a mitochondrial-dependent pathway.

### ENP-induced reproductive injury

As reviewed by Lan and Yang (2012) [11], some ENPs have the ability to penetrate the blood-testis barrier (BTB). The authors suggested that NP exposure may result in a generalized inflammation in the host and effect Leydig cells, causing reduced testosterone serum levels which then weaken the integrity of the BTB. In this regard the scale of the BTB gap became larger and allowed the NPs penetrating BTB easily. Both *in vivo* and *in vitro* studies have indicated that some ENPs exert toxic actions on male germ cells. ENP induced cell apoptosis was among one of the mechanisms of ENP's reproductive toxicity. According to Orazizadeh (2015) [65], mice receiving 300 mg/kg nano-TiO_2_ (ranging from 50 to 100 nm) for 35 days showed a significant decrease in testis weight, epidydimal sperm parameters, maturity of spermatogenesis and testosterone levels, with the apoptosis being demonstrated at each stage using the TUNEL method. Consistent with this was the increased expression of apoptotic related genes including *Bid*, *FasL*, *caspase-3* and *p38 MAPK*, implying an nano-TiO_2_ induced extrinsic pathway. Similarly, an *in vitro* study showed that male Leydig cells (TM3) and the Sertoli cells (TM4) exposed to 10 nm and 20 nm AgNP for 6 hours showed apoptosis, as evidenced by flow cytometry, and that this apoptosis could be partially mitigated by pretreatment of NAC, in accordance with the increased ROS levels in AgNP-treated cells [12]. The expressions of caspases 3, 8, 9 were all increased after AgNPs treatment, indicating that the apoptosis was induced both by intrinsic and extrinsic pathways. It should be noted that in the 10 nm AgNPs-treated groups, the phosphorylation of p38 and pErk1/2 was significantly higher than was that of control groups. However, in the 20 nm AgNPs treatment groups, the phosphorylation of Erk1/2, Bax, Bcl2 and RAD51 was increased. This suggests that the two different sizes of AgNPs induced cell apoptosis *via* two distinct signaling pathways.

Sperm are more sensitive to external environmental pollutants as they lack an effective enzyme defense system due to loss of cytoplasm during spermatogenesis [66]. Internalization of AgNPs (34-46 nm) into mouse spermatozoa resulted in decreased numbers of live cells, increased loss of mitochondrial copy numbers and other morphological abnormalities, as caused by AgNPs inducing ROS generation. The authors studied further and found that the AgNP-treated spermatozoa also resulted in poor fertilization and compromised embryo development. The ENP induced detrimental effects in sperm functionality was also observed in AuAPs-treated human sperm [15]. What surprises us is that neither sperm membrane nor sperm morphology was affected by the administration of 10.8 nm AuNPs or 7.8 nm AuNPs-oligonucleotide conjunction. Nevertheless, sperm motility and fertilization ability were significantly affected by the combination of AgNPs on the sperm membrane where toxic doses started at 10μg/mL.

## APOPTOSIS PATHWAYS INVOLVED IN ENP CYTOTOXICITY

**Figure 2:**
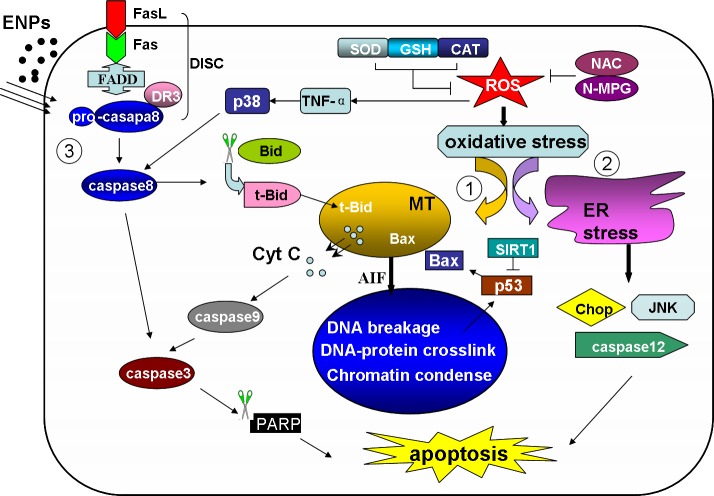
The ENP induced apoptosis pathways The ENPs induce two intrinsic pathways and one extrinsic death receptor pathway The ENPs generate excessive ROS, resulting in the peroxidation of lipids, protein and DNA. The oxidative stress then triggers mitochondria or ER induced apoptotic pathways. (1) Intrinsic mitochondria pathway. The oxidative DNA damage activates p53 and causes an increase in Bax levels. With the disruption of mitochondrial membrane potential the cytc and AIF is released to the cytoplasm. The cyt c together with caspase 9 and other factors form apoptosome and activate caspase 3, initiating cell apoptosis. The AIF is then translocated to the nucleus and induces DNA breakage. (2) Intrinsic ER pathway. The unfolded protein accumulation induces caspase 12 activation, and p38 MAPK, Chop and JNK are involved in this pathway. (3) Extrinsic pathway. The Fas association with FasL recruits FADD and pro-caspase8 forming DISC and activated caspase 8. Caspase 8 then activates caspase 3, or truncates Bid to tBid. tBid translocates into mitochondria and favors the release of Cyt C. MT: mitochondria. ER: endoplasmic reticulum. Cyt C: cytochrome c. DISC: death inducing signaling complex. tBid: truncated Bid.

Cells undergoing apoptosis may display blebbing, cell shrinkage, nuclear fragmentation and the appearance of apoptotic bodies along with protein denaturation, proteolysis and DNA fragmentation [67]. Apoptosis is a natural process that is important in embryogenesis, aging and in maintaining homeostasis of multicellular organisms. Deregulation of apoptosis can result in a variety of diseases; in the case of degenerative diseases apoptosis is overabundant, whilst in cases of malignancy it is over-suppressed. From the many studies listed above we can draw the conclusion that environmental ENPs are potential threats to human health *via* their ability to cause increased cell apoptosis in various normal organs. Apoptosis is also a well regulated aspect of cell death involving a complex interplay of organelles, molecules and signal transductions. The understanding of the specific mechanisms underlying ENP toxicity enables us to ensure their safe use or make specific modifications to either antagonize or mitigate the ENP-induced injury. According to previous studies, ENPs exposure results in cell apoptosis through a variety of pathways. These include ROS induced oxidative stresses, intrinsic mitochondrial pathways. ER-stress pathways and extrinsic Fas-FasL involved pathways (Figure 2).

Whilst the generation of ROS seems to be a common mechanism of ENPs, in some cases apoptosis is found, independent from oxidative stress, as induced by direct damage of organelles cause by ENP deposition [68]. For metal NPs, ROS generation may be caused by the release of metal ions. This has been proved in the case of AgNPs, nano-ZnO, CoO-NPs, etc. Other ENPs may be able to bind to proteins and affect their normal function as seen in the decrease of activities of antioxidant enzymes such as SOD, GSH, CAT and others. This results in an imbalance in oxidative status. Excessive ROS has been seen to result in a series of oxidative damages. These include protein and lipid peroxidation, the formation of DNA-protein crosslinks and DNA breakage. Protein oxidation is considered to be one of the main causes of cell death, resulting in accumulation of aberrant proteins and the loss of their function [69]. As cells are part of a huge membrane system, lipid peroxidation also results in a loss of membrane permeability and integrity. This is reflected by the increase in LDH level and disruption of mitochondrial membrane potential (Δψ) [64]. Oxidative DNA damage, if not repaired in time, is able to trigger the p53 induced cell G1- and S-phase arrest. P53 is also seen to be responsible for Bax upregulation and the release of cytochrome c following cell apoptosis.

ENP induced ROS triggers two apoptosis pathways, the intrinsic mitochondria and ER pathways. Under administration of ENPs, most cells show increased mitochondrial permeability and release of pro-apoptotic molecules such as cytochrome c and apoptotic inducing factor (AIF) into the cytoplasm. Cytochrome c release is regulated by various molecules such as t-Bid, Bax, and anti-apoptotic proteins like Bcl2. Cytochrome c, when released from mitochondria into the cytoplasm, is bound to caspase 9 and apoptotic protease activating factor 1 (Apaf1), forming a complex known as apoptosome which activates caspase 3 and initiates apoptosis [70]. AIF is translocated into the nucleus and causes DNA breakage into 5Х10^4^ fragments, inducing cell apoptosis in a caspase-independent pathway. Another ENP induced pathway, the unfolded protein response (UPR) pathway, is less common but also plays a role. In the UPR pathway, it is the alteration of intracellular Ca^2+^ concentration, accumulation of per-oxidative proteins and unfolded proteins triggers this ER pathway. Under normal conditions, the ER stress sensors are maintained in inactive state through association with a chaperone protein called glucose-regulated protein 78 (GRP78 or Bip), which dissociates when the aggregation of misfolded proteins as well as other stress events take place resulting in the initiation of UPR [71]. The UPR is considered to be a mitochondrial-independent and caspase 12-dependent pathway, which was activated along with the ER stress [72]. The activated caspase 12, together with other molecules, act as executors in the UPR-ER stress induced cell apoptosis. Although the specific mechanisms involved in UPR remain unclear, the p38 MAPK, JNK, Chop are all believed to be essential factors in this pathway.

ENPs were also able to activate Fas and FasL and trigger the extrinsic death receptor pathway. The combination of Fas and FasL recruits the adaptor protein FADD and pro-caspase 8, which together form the death inducing signaling complex (DISC) and activates caspase 8. Caspase 8, as an initiator caspase, cleaves and activates the executioner (caspase 3), leading to cell apoptosis and ultimately cell death. Caspase 8 is also able to cleave Bid into truncated Bid (tBid). This is then translocated into the mitochondria and favors cytochrome c release, initiating apoptosis in a mitochondrial-dependent pathway.

## ENP INDUCED APOPTOSIS IN CANCER CELLS

Direct induction of cell apoptosis by ENPs provides an opportunity for cancer treatment [66]. Among a variety of apoptotic stimuli, nanomaterials have been proven in numerous studies to be able to cause cancer cell apoptosis (Table 2).

**Table 2:** ENPs induced cancer cell apoptosis

ENPs type	ENPs size	Apoptosis in cancer cell	phenotype	Reference
AgNP	22 nm	human breast cancer cell line (MCF-7)	Cell shrinkage, nuclear condensation and fragmentation, DNA break up, apoptotic bodies	[73]
AgNP	Not mentioned	human lung carcinoma cell line A549	Increased Caspase 3/7/9 activities and mitochondrial depolarization, high levels of Bax, Smac/BIABLO,	[74]
AgNP	5 to 25 nm	human colon cancer cell (COLO 205)	chromatin condensation and nuclear fragmentation, MMP loss, increased ROS levels	[76]
AgNP	25 to 39 nm	human colon cancer cell(HCT-15, HT-29)	cell cycle arrest in G0/G1 phase	[75]
AgNP	7.5±2.5 nm	ovarian carcinoma cell line	DNA damage	[77]
,nano-Cr_2_O_3_	60 nm	Human lung cancer cell line A549	Increased ROS levels and antioxidant activities, caspase 3 activation	[38]
Au-Fe_2_O_3_ NPs	44.8±11.8 nm	human lung cancer HepG2 cells	shrinkage, budding and apoptotic body formation, increased ·OH and caspase 3 levels	[79]
Nano-TiO_2_	20-50 nm	A549	Increased ROS, decreased ATP	[78]
nano-HAP	<50 nm	human gastric cancer SGC-7901 cells	chromatin condensation and margination, cell blebbing and vacuoles, decreased mitochondrial membrane potential, release of Cyt C, increased caspase 3/9	[80]
nano-HAP	20 nm, 80 nm	human osteosarcoma MG-63 cells	nuclear fragmentation, formation of dense rounded apoptotic bodies, increased caspase 9	[81]
nano-HAP	<20 nm	mice leukemia P388 cells	irregular nucleus, ER expansion, mitochondrial swelling and collapse, cell arrest in G1 phase	[82]
MoO_3_ nanoplate	Width of 100-200 nm, length of 400 nm	human invasive breast cancer iMCF-7 cells	activated caspase 8 and caspase 9, increased ROS levels and declined mitochondrial membrane potential, condensed and fragmented chromatins	[84]
Nano-Se	59 nm	A357 melanoma cells	DNA fragmentation, phosphatidylserine translocation, oxidative stress, mitochondrial dysfunction	[85]
Realgar nanoparticles	80 nm	rat glioma C6 cells	Cell arrest in G0/G1 phases, increased Bax/Bcl2 ratio	[86]

### AgNPs induced cancer cell apoptosis

Silver nanoparticles (AgNPs), alongside some other metal nanoparticles, have been reported in a series of studies to have induced cancer cell apoptosis. Design, synthesis and manipulation of the AgNPs effective in this area has been a topic of increasing interest in recent years for potential applications in biology and medicine. The synthesis of AgNPs using plant extracts, for example, appears to be a simple, safe, effective as well as eco-friendly way to create an anticancer agent [73]. In one study, spherical shaped AgNPs with a size of 22 nm in a slightly agglomerated form were achieved by employing *Sesbanta grandiflora* leaf extract as reducing agent. When such kind of AgNPs were applied to human breast cancer cell lines (MCF-7) with concentrations of 20μg/mL for 48 hours, the treated cells displayed shrinkage, nuclear condensation and fragmentation as well as DNA break up characteristic of apoptosis. Apoptosis was then confirmed by the observance of apoptotic bodies. It was assumed by the authors that the AgNPs-induced ROS and the activation of caspase cascades were responsible for the DNA damage and the following apoptotic processes. However, more direct evidence is needed to confirm this. In another case, where AgNP was synthesized from aqueous leaf extract of *Albizia adianthifolia* (AA _AgNP_), the apoptosis pathways were investigated in detail using the human lung carcinoma cell line A549 [74]. It was found that caspase-3/−7 and casepase-9 activities were up-regulated in AA_AgNP_ -treated A549 cells as compared to controls. This was responsible for the cleavage of specific substrates leading to cell apoptosis or cell death. In addition, increased mitochondria depolarization, decreased ATP concentration and high levels of Bax expression were detected after AA _AgNP_ treatment, as accompanied by the release of the apoptotic regulator molecule Smac/BIABLO. This indicated an intrinsic apoptotic pathway induced by AA _AgNP_ in human cancerous lung cells. AgNPs synthesized by the crystal compound of *sodium para-hydroxybenzoate tetrahydrate* (SPHT), as isolated from *Vitex negundo.*L leaves, displayed a spherical shape and was arranged from 25 to 39 nm [75]. The SPHT-AgNPs were found able to exert an apoptotic effect on human colon cancer cell lines by IC_50_ inducing cell cycle arrest at the G_0_/G_1_ phase. Other biogenic-AgNPs from *Abutilon indicum* (AIAgNPs) were also presented in a spherical shape and had sizes ranging from 5 to 25 nm, as identified from the TEM imaging [76]. AIAgNP treated human colon cancer cell (COLO 205) at IC_50_ for 24 and 48 hours showed an orange-colored apoptosis body detected by AO/EB staining which was considered to be as a result of chromatin condensation and nuclear fragmentation. The apoptosis mechanism in AIAgNPs treated COLO 205 was attributed to the increased ROS levels and the loss of mitochondrial membrane potential (MMP). This, in turn, caused DNA fragmentation and cell cycle arrest.

Cancer cell apoptosis, as induced by AgNPs, has also involved other molecules. AgNPs with a size of 7.5±2.5 nm were reported to have induced ovarian carcinoma cell line apoptosis, as showed by increased DNA damage and increased apoptosis rate after AgNPs-treatment [77]. In this case, nuclear factor erythroid 2 (NF-E2)-related factor 2 (Nrf2) was proved to play a role in AgNPs-induced cell apoptosis where cells were more sensitive to AgNP-induced DNA damage and apoptosis in the Nrf2-RNAi treatment group as compared to the control. HO-1, one of the down-regulators of Nrf2, which engages the phosphoinositide 3-kinase (PI3K) as well as P38-MAPK pathways, was further proved to be active in cytoprotection against AgNP-mediated toxicity. In addition, elevated ROS levels and subsequent oxidative stress and the mitochondria mediated intrinsic pathways were shown to have been involved in most of the cases of ENP-induced cancer cell apoptosis.

### Other ENP induced cancer cell apoptosis

Some cancer cells can be induced cell death *via* different ENPs. The human lung cancer cell line A549, for example, displayed induced cell apoptosis *via* nano-Cr_2_O_3_ and nano-TiO_2_ exposure. The nano-Cr_2_O_3_ (60 nm) exposure to A549 cells resulted in a dose-dependent increase in ROS level and the activation of antioxidant defense systems. With caspase 3 activation, the decline in nano-Cr_2_O_3_ exposed A549 cell viability was assumed to be caused by apoptosis [38]. A549 cell viability was also significantly decreased after exposure to 50-300μg/mL nano-TiO_2_ for 4 hours. The nano-TiO_2_ was observed to be endocytosed by A549, causing a dose-dependent decrease of intracellular ATP levels which was related to the increased ROS level. This leads to cell apoptosis and cellular energy deficits [78]. In some cases the combination of two ENPs may provide, from the two differing components, special properties and excellent catalytic activities when used in combination. Gao et al. (2012) combined the 8 nm Au and 20 nmγ-Fe_2_O_3_ together with peptide conjunction and produced the 44.8±11.8 nm RGD/FITC-DEVD-Au-Fe_2_O_3_ NPs (DEVD as caspase 3 substrate) [79], which allowed the real-time imaging of caspase 3 induced apoptosis. The Au-Fe_2_O_3_ NPs of 30μg/mL were administrated to human lung cancer HepG2 cells, which initiated catalytic formation of hydroxyl radicals (·OH). After 6 hours, the HepG2 cells showed shrinkage, budding and apoptotic body formation with the maximum fluorescence intensity occurring at the highest caspase 3 level. The heterostructured NPs prove promising for future therapeutic applications in cancer.

Some ENPs have been reported to exhibit anti-cancer effects on several human cancers. Nanoscale hydroxyapatite (nano-HAP) was shown to have induced cell apoptosis in human gastric cancer SGC-7901 cells, osteosarcoma MG-63 cells, leukemia P388 cells, and others. Characteristic features of apoptosis such as chromatin condensation and margination, cell blebbing and vacuoles in SGC-7901 cells [80], nuclear fragmentation and formation of dense rounded apoptotic bodies in MG-63 cells [81], irregular nuclei, ER expansion, and mitochondrial swelling and collapse in P388 cells, were all observed beyond nano-HAP treatment [82]. Overall, mitochondria seem to be a main target of nano-HAP. The exposure of human gastric cancer SGC-7901 cells to 100μg/mL nano-HAP (smaller than 50 nm) for 12 hours caused decreased mitochondrial membrane potential, release of cytochrome c and activation of caspase 3 and 9. The caspase 9-dependent intrinsic mitochondrial apoptotic pathway also played a role in nano-HAP (20 and 80 nm) treated MG-63 cells. The cell arrest in the G1 phase of P388 cells caused by nano-HAP exposure (of less than 20 nm particle size) may be the result of disrupted mitochondrial morphology and function.

Nano structures of some essential elements were also explored as anti-cancer candidates. Molybdenum (Mo) is an essential trace metal in organisms and acts as a cofactor for various enzymes including aldehyde oxidase, xanthine oxidase, and sulfite oxidase [83]. MoO_3_ nanoplates with a width of 100-200 nm and a length of 400 nm were applied towards human invasive breast cancer iMCF-7 cells [84]. The MoO_3_ nanoplate exposure at 200 and 400μg/mL for 48 hours has resulted in elevated ROS levels and a declined mitochondrial membrane potential in iMCF-7 cells as well as condensed and fragmented chromatins, all of which suggest the presence of apoptotic bodies. With increases of activated caspase 8 and caspase 9, the MoO_3_ nanoplates might exert anti-cancer activities *via* both intrinsic and extrinsic apoptotic pathways. Another essential element, the selenium nanoparticle (Nano-Se), is known for its novel biological activity and low toxicity. The use of Nano-Se, with an average size of 59 nm, resulted in a broad spectrum of growth inhibition against A357 melanoma cells, Hep G2 hepatocellular carcinoma cells, MCF-7 breast adenocarcinoma cells and CNE2 nasopharyngeal carcinoma with IC_50_ values ranging from 3.0 to 14.1μM [85]. Further investigation on the mechanism suggested that the nano-Se treatment resulted in DNA fragmentation and phosphatidylserine translocation in A357 cells. In this, oxidative stress and mitochondrial dysfunction is thought to be involved in the nano-Se induced apoptosis pathway. Such results may render nano-Se as a candidate chemotherapeutic agent for human cancers.

High grade gliomas are among the deadest human tumors. ENPs, with their small sizes and ability to cross the BBB, have been used in an attempt by scientists to treat brain cancers. One example, realgar, has been used in China for thousands of years as a traditional Chinese medicine. According to An et al. (2011) [86], realgar nanoparticles with an average diameter of 80 nm have inhibited rat glioma C6 cell proliferation and induced apoptosis in a dose and time-dependent manner. Here, cell arrest in G0/G1 phases can be observed and the associated apoptosis may be caused by an increase in Bax/Bcl2 ratio. Realgar nanoparticles may therefore also provide a promising anti-cancer strategy and hopes towards utilization for human brain cancer therapy.

### ENP as a drug-delivery system for treatment of cancer

**Figure 3:**
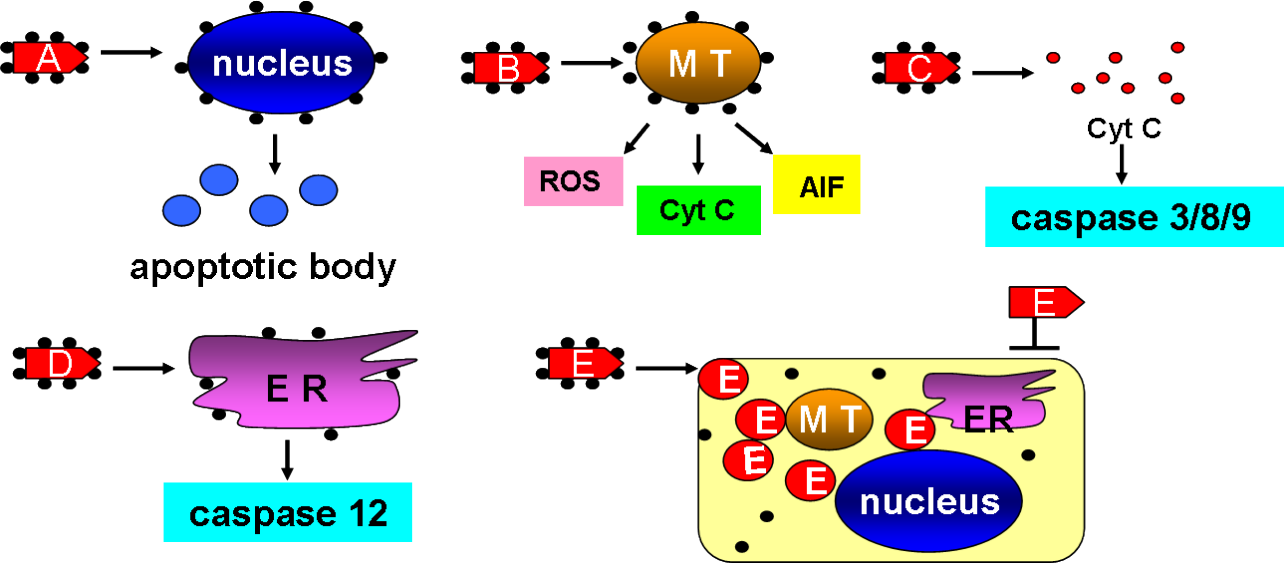
ENPs can be used as nano-carriers in cancer therapy **A.** ENPs were constructed with nucleus-targets and caused its deformation and induced apoptotic bodies. **B.** ENPs were constructed with MT-targets and caused ROS-dependent oxidative damages, cyt C-dependent apoptosis, and AIF induced nucleus breakage. **C.** ENPs were constructed with cyt C which were able to induce caspase 3/8/9-dependent apoptosis. **D.** ENPs were constructed with ER targets and induced caspase 12-dependent apoptosis. **E.** ENPs were constructed with cancer drugs that were not able to be taken into cancer cells. MT: mitochondria. ER: endoplasmic reticulum. cyt C: cytochrome c.

ENPs, with their unique properties such as surface charge, particle size, composition and surface modification with tissue recognition ligands or antibodies, has been increasingly explored as a tool to carry small molecular weight drugs as well as macromolecules for cancer therapy, thus generating the new concept “nanocarrier” [87]. Taking the brain as an example, the ENP nanocarriers applied to brain cancer include polymeric nanoparticles, liposomes, dendrimers, nanoshells, carbon nanotubes, superparamagnetic nanoparticles, nucleic acid based nanoparticles and antisense oligonucleotides [88]. There are several strategies that DNA, protein and drug molecules are constructed with ENPs, such as ionic interactions, covalent or non-covalent binding, absorption to surfaces, or *via* polymers like PEG and PEGylated phospholipids [89]. Here we review several nanocarrier examples targeting cancer cells and enhancing their apoptosis. Such a strategy includes various innovative opportunities for cancer therapy (Figure 3).

As nucleus damage, mitochondrial disruption and cytochrome c release all play a key role in regulating cell fates, each of these can be targeted to induce apoptosis in cancer cells. The nucleus, as the most important organelle in a cell, controls the growth, proliferation and apoptosis of a cell. Nuclear-targeted apoptosis, therefore, has been a clear goal for cancer therapy [90]. In one study nanoconstructs composed of nucleolin-specific aptamers and gold nanostars (Apt-AuNS, 25 nm) were selectively targeted to nucleus of Hela cells. This leads to the deformation of the nuclear envelop in over 60% of Hela cells with nanoconstructs after 7 h exposure [91]. Such a nuclear deformation may be caused by the release of aptamers from the surface of gold nanostars upon delivery. Beyond administration the nuclear phenotype and the biological activity of caspase 3 and 7 were found to be correlated, with more nuclear deformation and increased caspase 3 and 7 as well as less cell viability. Mitochondria are also an attractive target for the design of effective and specific cancer therapeutics. Mkandawire et al. (2015) had AuNPs (20 nm) conjugated to a variant of turbo green fluorescent protein (mito TGFP) that harbored an amino-terminal mitochondrial localization signal. He then transfected the AuNPs conjugates into the human breast cancer cell line Jimt-1 [92]. The mitoTGFP-AuNPs conjugates were directed to mitochondria upon transfection, causing the rupture of the mitochondrial outer membrane, enabling the conjugates to make their way to the inner membrane. This, in turn, released cytochrome c led to mitochondrial-dependent apoptosis as evidenced by cell shrinkage and condensed chromatin in mitoTGFP-AuNPs including in the Jimt-1 cells. In the study of Morales-Cruz et al. (2014), a cytochrome c drug delivery system was constructed to induce apoptosis in Hela cells. Cytochrome c was made nanosized into 100 to 300 nm using a solvent-displacement method, the particle surface of which was decorated with poly (lactic-co-glycolic) acid-SH *via* a linker to prevent premature dissolution during delivery. When incubated with 25 to 100 μg/mL cytochrome c NPs for 6 hours, the Hela cells showed significant decrease in viability where more than 70% of cells were dead in the 100 μg/mL group and apoptosis was confirmed by co-localization of DAPI and PI [93].

It is well known that tumor cells can be resistant to chemotherapeutics. With the assistance of nanoparticles, medical treatment can advance more smoothly. Polysaccharides, as major bioactive compounds, are reported to have anti-cancer properties. They have been able to induce cell death in a hepatoma cell lines (Hep G2) [94]. Here, Nano-encapsulated in chitosan-silica or just silica, the nano-conjunction caused a more significant decrease in Hep G2 cell viability than mere polysaccharides alone and induced apoptosis like ROS generation, DNA fragmentation, caspase activation and cell sub-G1 phase arrest. In another case, daunomycin, one of the most important antitumor drugs clinically applied in acute leukemia, was loaded with magnetic nanoparticle Fe_3_O_4_ or Au to facilitate anti-cancer activity in K562/A02 cells [95]. Breast cancer remains a threat to women's health worldwide. Anti-metadherin (anti-MTDH) antibodies which are specific to breast cancer cells, were bound to AuNPs *via* [^18^F]2-fluoro-2-deoxy-d-glucose (18F-FDG) [96]. The ^18^FDG-AuNPs-Anti-MTDH conjunction was co-incubated with breast cancer MCF7 cells and an apoptosis rate of 20% was observed, compared with the 2% in the control. In the study of Li et al. (2014), a brain metastatic breast cancer model was established in mice where doxorubicin (Dox) was loaded to PMAA-PS 80-g-St nanoparticulates. The Dox-loaded nanoparticles (10 mg/kg Dox) were injected into the breast cancer brain metastasis models for 2 weeks, during which the Dox NPs managed to cross the BBB and accumulated in the intracranial breast cancer brain metastases. There it significantly inhibited the growth of tumors as compared to free Dox treatment. It may be that the released Dox induced cancer cell apoptosis through interference with DNA repair and replication by DNA intercalation in highly proliferating cells. In such a process normal cells may be spared [97]. ENPs are also explored as small interfering RNA (siRNA) carriers in the treatment of breast cancer [98], ovarian cancer [99], hepatocellular carcinoma [100], and B-cell malignancies [101] etc., as methods in gene silencing therapy. The above examples show that ENP-dependent drug delivery systems prove to be a promising therapeutic system for the treatment of cancer.

Despite the promising drug delivery system provided by ENPs in cancer therapy, one has to consider the safety and efficiency of the delivery and targeting. To achieve selective treatment and to reduce toxicity, ENPs are usually endowed with tumor targeting abilities by binding to antibodies directed against highly expressed cancer cell surface receptors. The drugs may be encapsulated within ENPs and then conjugate to targeting antibodies (Antibody-Nanoparticle Conjugates, ANPs) [102]. For example, α-Hed, which induced cell death mainly *via* apoptosis, was conjugated with chitosan nanoparticles (CS-NP) to form α-Hed-CS-NPs in one study [103]. The α-Hed-CS-NP surface was then modified with monoclonal antibody CD147 and formedα-Hed-CS-CD147-NPs with sizes ranging from 50 to 300 nm, which showed a higher cellular uptake and intracellular accumulation in human liver cancer cell lines HepG2 and SMMC-7721 than free α-Hed and α-Hed-CS-NPs, and also had higher targeting antitumor efficacy at the tumor sites in nude mice. In another case the melanotransferrin antibody (MA) was conjugated with solid lipid nanoparticles (SLNs) to carry anticancer etoposide (ETP) across the BBB for managing glioblastoma multiforme (GBM) [104]. MA was crosslinked on the surface of etoposide-loaded SLNs (ETP-SLNs) to form MA-ETP-SLNs, which were shown to be appropriate for the transport of etoposide across the BBB and be able to inhibit the growth of GBM, indicating a potential application in the colloidal delivery system for malignant GBM pharmacotherapy. In the newly published literature of Palanca-Wessels et al. [105], the authors introduced the usage of linking an internalizing streptavidin-conjugated HER2 antibody to an endosome-disruptive biotinylated polymeric nanocarrier for the functional cytoplasmic delivery of siRNA in breast and ovarian cells, resulting in an 80% reduction of target mRNA and protein levels with sustained repression for no less than 96 hours. Plus, the effective targeting can be realized by designing smart nano-carriers that respond to certain changes in the bio-environment and release the encapsulated contents on demanded sites. Gurka et al. [106] designed a dual targeting system in mesoporous silica nanoparticle (MSN) in the adventure of pancreatic cancer treatment, taking advantage of the overexpressed tumor receptor urokinase plasminogen activator receptor (UPAR) as well as the acid tumor microenvironment. It was found that the tumor specificity of MSN was improved with the addition of chitosan (targeting acid PH) and urokinase plasminogen activator (UPA, targeting UPAR), ensuring drug release and accumulation preferentially at the pancreatic tumors compared to liver or kidney. Targeted as well as controlled drug delivery and release can also be realized with magnetoelectric nanoparticles (MEN), which, with its load, can be controlled and placed at the intended site *via* application of an external magnetic field [107].

## CONCLUSIONS AND PERSPECTIVE

ENPs, having great potential for application in cancer treatment, also arouse concerns about potential risks for human exposure. How to use ENPs wisely at minimum side effects is becoming an increasingly important focal area. Several particle features, such as type, size, shape, zeta potentials, dispersion/agglomeration status and surface modification should be taken into account in evaluating ENP safety. Here we introduce several examples concerning ENP type, size and shape as well as surface modification.

### ENPs type-dependent cytotoxicity

Before application, the comparable cytotoxicities between many differing particles must be established by comparing their effects in different cell lines. In one case six different fine- or nano- particles were investigated on the macrophage-like murine cell line RAW 264.7. The particles were crystalline silica (DQ12, 960 nm), amorphous silica (a-SiO_2_, 14 nm), TiO_2_ ultrafine (uf TiO_2_, 20-80 nm), TiO_2_ fine (f TiO_2_, 40-300 nm), ZnO (10 nm) and MgO (8 nm). It was demonstrated that ZnO, DQ12 quartz and a-SiO_2_ were cytotoxic whilst toxicity was not in evidence for MgO, or fine and ultrafine TiO_2_, as referred to apoptotic effects for hypodiploid DNA in caspase 3/7 activation and effects upon cell live/death rates [108]. In another report, silver particles of nano-(20 nm) and submicron- (200 nm) were compared with TiO_2_-NPs (21 nm) in the human testicular embryonic carcinoma cell line Ntera2 and primary testicular cells from C57BL6 mice. The results showed that the silver particles exerted more of a cytotoxic effect than did the TiO_2_-NPs, causing apoptosis, necrosis and decreased proliferation in dose and time dependent manner [109]. TiO_2_ NPs seemed to be less toxic than other particles, though case by case analyses remain necessary.

### ENPs size and shape-dependent cytotoxicity

ENP size has been generally considered the most dominant factor for ENP cytotoxicity. In general, the smaller the ENPs size, the more concerns there is for human exposure. This may be due to the effect of mass-specific surface area, leading to the smaller sized ENPs exerting a more toxic effect. The bioavailability of nano- and microscale CuO (CuO NP and CuO MP) were explored in A549 and Hela S3 cells, as compared with CuCl_2_ [110]. A high cytotoxicity of CuO NP (55 nm) and CuCl_2_ were found, while the CuO MP (1289 nm) cytotoxicity was not in evidence up to 50μg/mL. In this, CuO NPs were seen to exert the strongest effects. However, small particles do not necessarily lead to better uptake or enhanced toxicity. In a NT2 study, for example, primary testicular cells were treated with nano-(20 nm) and submicron-(200 nm) Ag particles. Here it was the 200 nm AgNPs that caused more DNA damage (20%) than 20 nm AgNPs (15%) under same conditions [109]. Another study by Park (2007) reconfirmed that by investigating different cell fates induced by different sizes of TiO_2_ nanotubes, in which cellular activities were enhanced by particles less than 30 nm while reduced by particles larger than 50 nm with a high extent of apoptosis [111].

In addition to size, the shapes of ENPs are also directly correlated to their induced cytotoxicity. Two graphitic nanomaterials, graphene layers (G) and single-wall carbon nanotubes (SWCNT), having similar chemical composition and crystalline structures, were presented with different shapes. Here, G occurs in flat atomic sheets and nanotubes are tubular. In the concentration-dependent toxicity testing of these two ENPs on PC12 cells, results were dissimilar. G had a higher toxic effect at lower concentrations and SWCNT a more intense toxic effect at higher concentrations. This indicates different toxic mechanisms related to shape [112]. These different effects were assumed to be that the needle-like CNTs were more mobile and more easily able to penetrate the cell membrane and thereby caused strong interactions with various protein systems. G cytotoxicity may instead be correlated with the aggregation/agglomeration forms found on cell membranes. The indication is that these two ENPs have different cellular target sites. In another case four types of hydroxyapatite nanoparticles (nano-HAP) with different nanocrystal morphologies (short rod-like, long rod like, spherical and needle-shaped crystals) and sizes (10-20, 10-30, 20-40 and 20-40 nm) were compared for their effects upon primary cultured rat osteoblasts [113]. These nano-HAPs induced mitochondrial and caspase dependent apoptosis in the osteoblasts, with needle-shaped and the spherical particles inducing the greater cellular injury than short rod-like and long rod-like particles. It has been recognized that the specific surface area of ENPs can determine the activities of the materials. Particles with higher specific surface area tend to have greater activity and more easily attach to the cells and lead to toxicity. This is in accordance with the above study in which the needle-shaped (148.140 m^2^/g) and the spherical (122.479 m^2^/g) particles had larger specific surface areas than the short rod-like (45.002 m^2^/g) and long rod-like (68.452 m^2^/g) particles.

### ENPs surface-dependent cytotoxicity

ENPs of different surface modifications have numerous technological and biomedical applications. They interact with biological structures and display distinct and specific impacts to the cell systems. Graphite nanoparticles (GO) with a lateral width of 200-500 nm and a thickness of 1 nm were modified in different ways including the modification of their thickness, LA-PEG-GO (2 nm), PEG-GO (1.9 nm) and PEI-GO (2.5 nm). The modified and unmodified GOs were then co-incubated with human lung fibroblast cells (HLF). It was found that exposure to GOs led to mitochondrial dysfunction and increased the amount of HLF apoptotic cells, while values of the cell viability rates and DNA damage differed among the various modifications. This may be a result of different electronic charges on the surface of GOs after modification (−65.1mV for GO, +18.4mV for LA-PEG-GO, −8.86mV for PEG-GO and +60.5mV for PEI-GO) [114]. The electronic charge on the surface of the ENPs may play important roles in determining their toxicity to targeted cells. Similarly, Arvizo et al. (2010) investigated the different surface charge of AuNPs on various cell lines including malignant cells of ovarian cancer CP70 and A2780, nonmalignant cells of human bronchial epithelial cell BECs and human airway smooth muscle ASM cells, (24.4 mV for +AuNP, −1.09 mV for 0AuNP, −37.9 mV for −AuNP, and −1.94 mV for ±AuNP) [115]. The results showed that the uptake of +AuNP was significantly higher than other charges of AuNPs across different cell lines and that such an uptake induced membrane depolarization and altered intracellular Ca^2+^ concentrations leading to the inhibition of the cell proliferation of BECs and cell apoptosis of both CP70s and BECs.

Some modifications might be able to lighten the cytotoxic effects of ENPs. These modifications were supposed to improve their solubility and biocompatibility and alter their cellular interaction pathways, resulting in an enhanced ability to penetrate biological membranes with relatively low cytotoxicity [89]. Polystyrene nanoparticles of about 110 nm diameter were surface functionalized with carbonxyl (PS-COOH) or amino (PS-NH_2_) groups and were applied to macrophages of THP-1 leukemia cells [116]. Whilst the PS-COOH did not affect TP1 cell proliferation or exhibit any toxic effects on the macrophages, PS-NH_2_ particles not only inhibited THP-1 proliferation but also induced cell apoptosis in THP-1 cells. Carbon-based substances were considered to be less bio-reactive in biological systems and thus be critical for reducing the inherent reactivity of ENPs with biomolecules. In another case, nano-TiO_2_ was coated with polyacrylate and the cytotoxicity on Chinese hamster lung fibroblast (V79) cells was compared with nano-TiO_2_ and micro-TiO_2_. Here, the cell viability decreased and induction of apoptosis was observed for the administration of all types of TiO_2_ particles, but for polyacrylate-coated nano-TiO_2_, it was only detected in higher concentrations with no DNA damage detected [117].

## SUMMARY

Nanoparticles offer a numerous application possibilities in the industrial sector. These include their use as fuel additives for catalysis, as sunscreen additives for UV protection, various applications in the textile industry and their use in biomedicine as drug targeting agents or drug carriers [100]. In recent years directs ENP applications towards cancer therapy for inducing cancer cell apoptosis has been an increasing focus. Unfortunately such widely usage may also pose unwanted threat to human health. This calls for the necessity of a precise analysis of ENP cytotoxicity in living cells and the understanding of how their exact properties (size, shapes, surface charges, dispersion/agglomeration status) all play a deciding ENP safety and suitability for such roles. In addition, some aspects of surface modification may be able to reduce the bio-reactivity of ENPs, thus alleviating their toxicities in certain circumstances. This may provide a way to design even more effective particles of minimum undesired toxicity.

**Table:** 

Abbreviations	Full name
NPs	nanoparticles
ENPs	engineered nanoparticles
BBB	blood-brain barrier
BTB	blood-testis barrier
CNS	central nervous system
AgNPs	silver nanoparticles
CNTs	carbon nanotubes
ROS	reactive oxygen species
TEM	transmission electron microscopy
SEM	scanning electron microscopy
ER	the endoplasmic reticulum
SIRT1	sirtuin 1
HaCaT	human keratinocyte cells
FADD	Fas-associated proteins with death domain
DISC	death including signaling complex
AIF	apoptosis-inducing factor
PC12	dopaminergic neuron cells
GSH	glutathione
MDA	methane dicarboxylic aldehyde
SOD	superoxide dismutase
N-MPG	N-(mercaptopropinyl)-glycine
t-Bid	Truncated-Bid
MAPK	mitogen-activated protein kinase
JNK	cJun N-terminal kinase
LDH	lactate dehydrogenase
CAT	catalase
H2O2	hydrogen peroxide
CHOP	C/EBF-homologue protein
UPR	unfolded protein response
DISC	death inducing signaling complex
Nrf2	nuclear factor erythroid 2 (NF-E2)-related factor 2
cyt C	cytochrome c
